# Extremely strong tubular stacking of aromatic oligoamide macrocycles†
†Electronic supplementary information (ESI) available. See DOI: 10.1039/c4sc02380c


**DOI:** 10.1039/c4sc02380c

**Published:** 2014-09-16

**Authors:** Mark A. Kline, Xiaoxi Wei, Ian J. Horner, Rui Liu, Shuang Chen, Si Chen, Ka Yi Yung, Kazuhiro Yamato, Zhonghou Cai, Frank V. Bright, Xiao Cheng Zeng, Bing Gong

**Affiliations:** a Department of Chemistry , the State University of New York at Buffalo , Buffalo , New York , USA 14260 . Email: bgong@buffalo.edu ; http://www.chemistry.buffalo.edu/people/faculty/gong/; b Department of Chemistry , University of Nebraska-Lincoln , Lincoln , Nebraska 68588 , USA; c X-ray Science Division , Argonne National Laboratory , 9700 South Cass Avenue , Argonne , IL 60439 , USA; d College of Chemistry , Beijing Normal University , Beijing 100875 , China

## Abstract

As the third-generation rigid macrocycles evolved from progenitor **1**, cyclic aromatic oligoamides **3**, with a backbone of reduced constraint, exhibit extremely strong stacking with an astoundingly high affinity (estimated lower limit of *K*_dimer_ > 10^13^ M^–1^ in CHCl_3_), which leads to dispersed tubular stacks that undergo further assembly in solution. Computational study reveals a very large binding energy (–49.77 kcal mol^–1^) and indicates highly cooperative local dipole interactions that account for the observed strength and directionality for the stacking of **3**. In the solid-state, X-ray diffraction (XRD) confirms that the aggregation of **3** results in well-aligned tubular stacks. The persistent tubular assemblies of **3**, with their non-deformable sub-nm pore, are expected to possess many interesting functions. One such function, transmembrane ion transport, is observed for **3**.

## Abstract 2

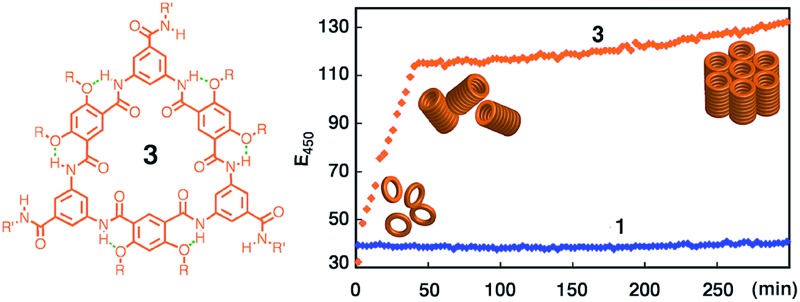
Aromatic oligoamide macrocycles 3 undergo extremely strong stacking in both solution and the solid state, forming tubular assemblies that further aggregate.

## Introduction

Tubular structures, with their cylindrical shapes, defined outer and inner surfaces, and internal pores, provide versatile structural modules for constructing functional structures.^1,2^ Nanopores with precisely defined diameters of less than 2 nm, such as those of carbon nanotubes (CNTs), exhibit many fascinating properties.^3,4^ Compared with carbon nanotubes, organic nanotubes^5^ have unique advantages such as ready functionalization, versatile compatibility, and modular assembly. Among known strategies,^2,5,6^ the superposition of cyclic building blocks^5a,e–g,7^ represents an approach that combines the ready modifiability of small molecules and the power of self-assembly, leading to nanotubes with structural and functional tunability. However, the alignment of cyclic molecules based on non-covalent forces is often impeded by limitations such as the poor directionality of π–π stacking and/or the limited strength of hydrogen-bonding, especially in polar media, which frequently lead to undesired outcomes upon even a slight structural modification on an otherwise promising building block.

Given the many remarkable functions exhibited by or expected of non-deformable nanopores,^3,4^ organic nanotubes resulted from the stacking of rigid macrocycles, which contain non-collapsible inner pores, is especially attractive.^8^ While many rigid macrocycles such as those with π-conjugated^9,10^ and other backbones,^11–15^ along with tubular stacks of some of these macrocycles in the solid and liquid crystalline phases,^5d–f,7^ are known, self-assembling nanotubes that stably exist in solution are rare. The availability of stable nanotubular assemblies should greatly advance the development of systems with properties typically associated with biological structures. Achieving this objective requires the strong, directional stacking of cyclic building blocks.

Over the years, we have developed several different classes of rigid macrocycles containing non-deformable internal cavities.^16^ The first generation of such molecules are aromatic oligoamide macrocycles **1**,^14*a*^ which were found to form efficiently in one pot while we attempted to prepare folding aromatic oligoamides^17,18^ and polyamides^19^ having similar backbones. The one-pot macrocyclization we found has led to rigid macrocycles containing internal cavities of 5 to 30 Å across.^16,20^ The benzene residues of macrocycles **1** are connected *via* amide groups engaging in highly favourable three-centre intramolecular hydrogen-bonding interaction^21^ that constrains the macrocyclic backbones. With fully constrained, non-deformable backbones, macrocycles **1** were observed to strongly aggregate in solution and form tubular stacks in the solid state.^22^

To better control the alignment of these molecules, amide side chains are attached to **1**, which led to the second-generation macrocycles **2**. Being flanked by alkoxy side chains, the amide side chains of **2** are perpendicular to the benzene rings to which they are attached and should thus be predisposed to intermolecular H-bonding that enforces the macrocycles to stack on top of one another into a tubular stack. Surprisingly, studies using multiple analytical techniques suggested that macrocycles **2** underwent insignificant aggregation.^23^ It seemed that the amide side chains of **2** not only failed to engage in intermolecular H-bonding, but also abolished the otherwise strong aggregation of **1**.
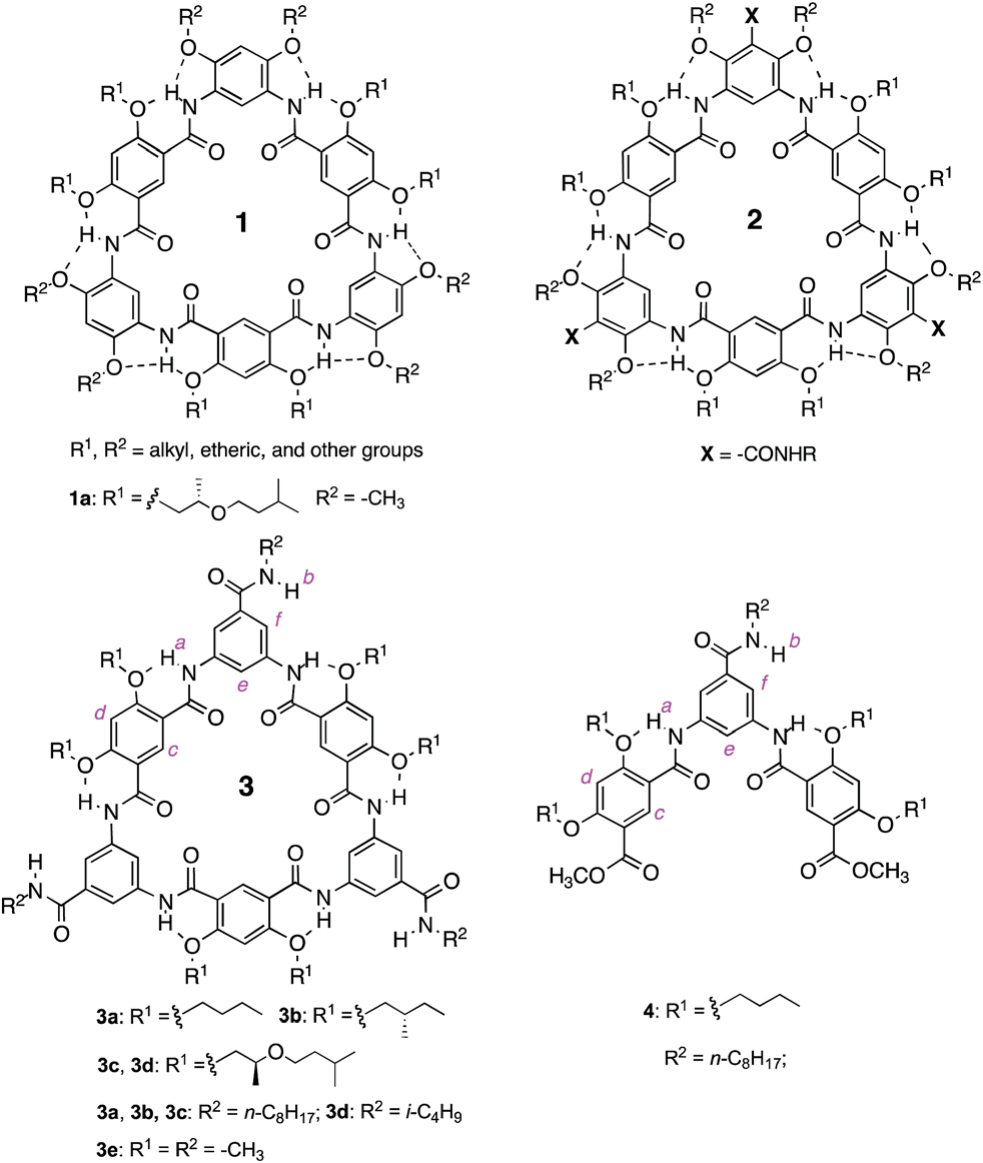


The unexpected behavior of macrocycles **2** may be due to steric crowdedness that hampers side-chain H-bonding and backbone π–π stacking. Such a besetment could be evaded by removing the alkoxy groups flanking the amide side chains of **2**, which leads to **3**, our third-generation aromatic oligoamide macrocycles.^24^ Herein, we report the unusually strong tubular stacking of **3**. It was found that, in solution, macrocycles **3** underwent aggregation that was mediated by their backbones and weakened by polar solvents. The self-association of **3** is extremely strong, with a strength that is, to the best of our knowledge, unprecedentedly high. The strong association of **3** results in individually dissolved columnar stacks that dominate at low concentrations and further pack at elevated concentrations. X-ray diffraction (XRD) revealed the columnar stacks of **3** and their hexagonal packing in the solid state. Consistent with their reliable tubular self-assembly, macrocycles **3** were found to mediate efficient transmembrane transport of ions.

## Results and discussion

### Backbone-mediated aggregation

The aggregation of macrocycles **3a–d** was first indicated by their ^1^H NMR spectra. In CDCl_3_, no signals could be found in the amide and aromatic region. The only peaks observed are those from 0.5 ppm to 1.8 ppm, which belong to the terminal alkyl groups of the side chains (Fig. S1 in the ESI†). This observation suggests that **3a–d** undergo decreased molecular motion due to aggregation involving their oligoamide backbones. In DMF-d_7_ or DMSO-d_6_, the ^1^H NMR spectra of **3a–d** contain well dispersed signals (Fig. S2†), suggesting that the aggregation of these molecules is interrupted in polar solvents.

**Fig. 1:**
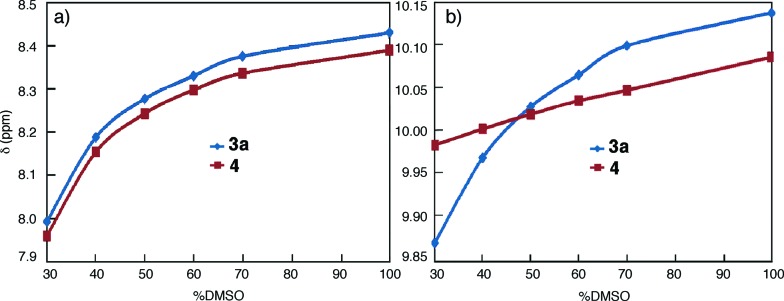
Plots of the chemical shifts of (a) protons *b* and (b) protons *a* of **3a** (1 mM, blue) and **4** (1 mM, red), against volume percent DMSO-d_6_ in CDCl_3_.

To gain additional insights, the ^1^H NMR spectra of **3a** were recorded in CDCl_3_ (1 mM) containing incremental proportions of DMSO-d_6_. The signals of amide and aromatic protons only become obvious in solvents with 30% or more DMSO-d_6_ (Fig. S3†). The same trend was observed with increasing ratios of DMF-d_7_ (Fig. S4†). In comparison to macrocycles **1** that gave well dispersed ^1^H NMR signals with as few as 5% DMSO-d_6_ or DMF-d_7_ in CDCl_3_,^14*a*^ the aggregation of **3a**, as indicated by the effect of DMF or DMSO, is much stronger. Plotting the chemical shifts of the amide protons of **3a** and those of **4** against DMSO-d_6_ contents indicates that amide protons *b* of **3a** and **4** follow the same trend with changing solvent polarity (Fig. 1a). This observation suggests that the side-chain NH groups of **3a**, like that of the molecularly dissolved **4** (Fig. S5†), are exposed to solvent. In contrast, with increasing ratios of DMSO-d_6_, the downfield shifts of amide protons *a* are non-linear for **3a**, and linear for trimer **4** (Fig. 1b). The different behavior of protons *a* of **3a** and **4** can be explained by the stacking of **3a** in CDCl_3_, which shields the oligoamide backbone from solvent molecules. Increasing solvent polarity weakens and eventually breaks up the aggregates, which exposes individual molecules, and hence protons *a*, of **3a** to solvent molecules.

Experiments based on diffusion-ordered spectroscopy (DOSY) in CDCl_3_ containing 40% DMF-d_7_ clearly demonstrated the aggregation of **3a** and the lack of aggregation in pure DMF-d_7_ (Fig. S6†). Dynamic light scattering (DLS) was also used to compare the aggregation of **3a** and **1a**. In DMF, neither **3a** nor **1a** formed any noticeable aggregate. In contrast, aggregates of **3a** (1 mM), with an average hydrodynamic diameter [(2.6 ± 0.6) × 10^4^ nm] that is much larger than that [(250 ± 18) nm] of the aggregates formed by **1a** were observed in chloroform. The DLS results corroborate those from DOSY and ^1^H NMR, confirming that the aggregation of **3a** is much stronger than that of **1a**.

### Ground-state aggregation

**Fig. 2:**
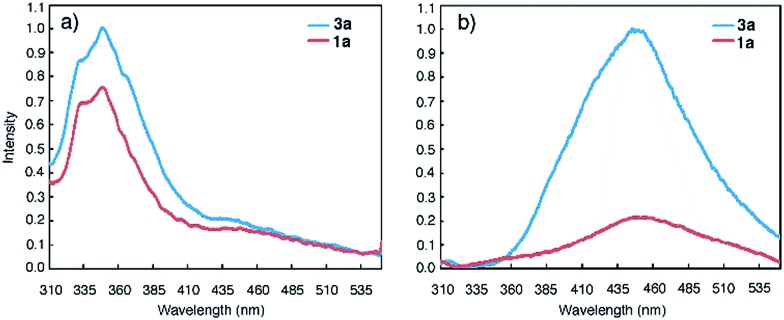
Fluorescence spectra of **1a** (red) and **3a** (blue) in (a) DMF (1 μM) and, (b) CHCl_3_ (1 μM) using an excitation wavelength of 282 nm.

Macrocycles **1a** and **3a** were then examined at 1 μM, a concentration that is three orders of magnitude lower than those used for NMR and DLS studies, with fluorescence spectroscopy. In DMF, emission bands centred at 350 nm, which can be ascribed to molecularly dissolved monomers, are observed (Fig. 2a). In CHCl_3_, macrocycles **1a** and **3a** display broad, red-shifted bands at 450 nm (Fig. 2b). The 450 nm bands are reminiscent of excimer fluorescence typical of π-stacked aromatic rings^25^ that exist in the ground state (*i.e.*, due to aggregation) and give “excimer-like” emission.^26^ Consistent with the ground-state association of **3a**, monitoring the emission bands of **3a** (125 nM and 0.1 pM in CHCl_3_) at 350 nm and 450 nm revealed two different peaks at 260 nm and 280 nm, respectively, in the excitation spectra (Fig. S7†).

**Fig. 3:**
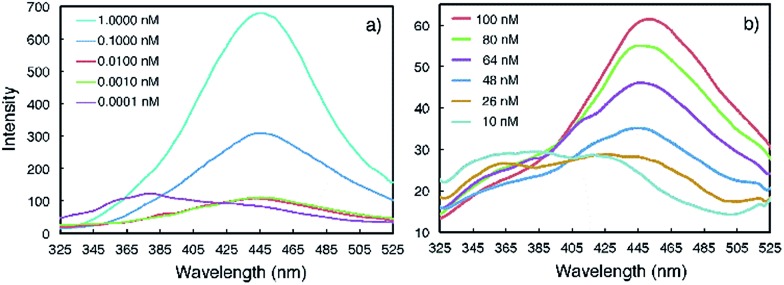
Fluorescence spectra of (a) **3a** and (b) **1a** recorded at different concentrations in CHCl_3_ using an excitation wavelength of 282 nm.

Emission spectra collected at reduced concentrations in CHCl_3_ indicate that **3a** remains aggregated down to 1 pM and exists as monomers only at 0.1 pM (Fig. 3a). Assuming that, at 1 pM, macrocycle **3a** is involved in a monomer-dimer equilibrium^10*b*^ with a 10% dissociation, a lower limit of 4.5 × 10^13^ M^–1^ for the dimerization constant can be estimated, which suggests that **3a** engages in remarkably strong association. In contrast, the fluorescence spectra of **1a** recorded below 100 nM contain emission bands at both 350 nm and 450 nm; at 10 nM, the emission band at 450 nm greatly weakens while the one around 350 nm becomes dominant (Fig. 3b). These observations demonstrate that the aggregation of **3a** is several orders of magnitude stronger than that of **1a**.

The effect of solvent polarity on the aggregation of **3a** and **1a** (1 μM) was revealed by plotting *E*_450_/*E*_350_, the ratios of normalized fluorescence emissions at 450 nm and 350 nm, as a function of volume percent CHCl_3_ in DMF (Fig. S8 and Table S1†). The *E*_450_/*E*_350_ ratio rises with increasing volume percent CHCl_3_ in DMF. In contrast, the intensity of the 450 nm band of **1a** (1 μM) is much less prominent than that of **3a**. These observations confirm the high sensitivity of the aggregation of **3a**, even at a very low concentration, to solvent polarity, which implies the involvement of a strong dipole–dipole factor in the self-association of this compound.

### Insights from computational study

To gain insights into the strong self-association of **3**, *ab initio* computation was performed on a dimer consisting of two stacked molecules of model macrocycle **3e**. The potential energy as a function of the relative stacking angle between two such macrocyclic units was calculated at the density-functional theory (DFT) level of M06-2X/6-31G(d), with the molecular structure being optimized at the DFT BLYP-D3/GTH level with inclusion of dispersion correction (see the ESI†). It was found that the dimer with a stacking angle of 60.5° gave the strongest binding, with a binding energy of –49.77 kcal mol^–1^. In contrast, the dimer involving two “eclipsed” molecules, *i.e.*, with a stacking angle of 0°, had a binding energy of –24.42 kcal mol^–1^. The drastically enhanced stability of the most stable dimer may be explained by the highly cooperative interaction of local dipoles. With a stacking angle of 60°, the two different types of benzene residues, one derived from the diacid monomer and the other derived from the diamine monomer, stack directly on top of each other and undergo favourable dipole–dipole attraction. The highly cooperative action of six such pairs of benzene residues is most likely responsible for the observed strong association of **3a**.

### Time course of aggregation: a two-stage process

**Fig. 4:**
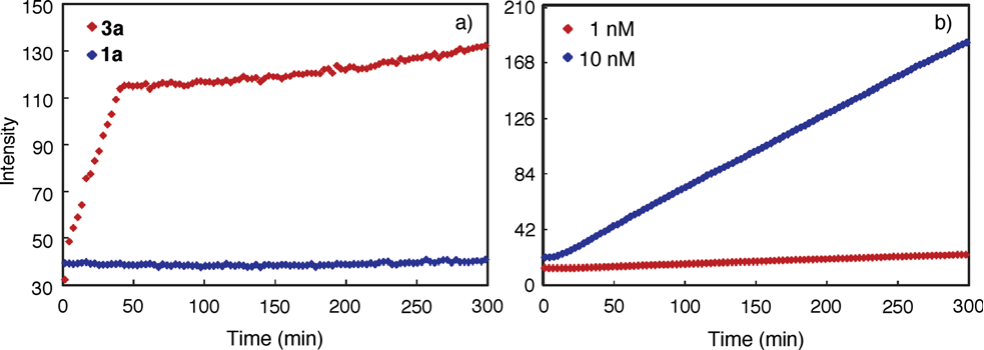
Change of fluorescence intensity at 450 nm as a function of time. (a) Upon mixing **3a** or **1a** dissolved in DMF with CHCl_3_. The final concentration of **1a** or **3a** is 1 μM and, (b) **3a** (10 nM and 1 nM), in the mixed solvent of CHCl_3_ and DMF (1/1, v/v). The measurements were carried out using an excitation wavelength of 281 nm.

The progress of the aggregation of **1a** and **3a** was monitored by following the intensity of the 450 nm band upon adding a solution of **3a** or **1a** in DMF into CHCl_3_. It was found that the rate of aggregation increased with increasing proportion of CHCl_3_ (Fig. S9†). Fig. 4 shows the time courses for the 450 nm band of **1a** or **3a** (1 μM) in CHCl_3_ and DMF (1/1, v/v). The aggregation of **3a** involves two stages: an initial rapid growth phase that lasts for about 37 min, followed by a much slower growth phase (Fig. 4a, red). In the same solvent, the aggregation of **1a** is negligible, with no obvious increase being observed for its 450 nm band (Fig. 4a, blue). Lowering the concentration of **3a** decreased the rates of aggregation considerably (Fig. S10†) and, below certain concentration, resulted in the disappearance of the second growth phase, even at greatly increased CHCl_3_ content. For example, at 10 nM, macrocycle **3a**, being aggregated as shown by its 450 nm band (Fig. S11†), gives one growth phase (Fig. 4b). At 1 pM in the same solvent, macrocycle **3a** exists mainly as monomers (Fig. S12†) and, consistent with the lack of aggregation, shows insignificant increase of emission at 450 nm (Fig. S13†).

The observed fluorescence emission and two-stage time course associated with the aggregation of **3a** may be rationalized by a model that involves an initial (fast) assembling period during which the molecules of **3a** undergo strong, backbone-mediated stacking, followed by a second (slow) phase in which the columnar stacks of **3a** further pack *via* the surface interactions between columns (Fig. S14†). The initial phase is fast because, when **3a** starts to aggregate, the concentration of monomer is high and that of the columns is negligible. The packing of columns is slower because the concentration of the columns is much lower than the monomers and it takes more time for the columns to diffuse and then to achieve optimum surface contact. The sharp transition from the first to the second phase thus indicates a threshold beyond which the packing of columns becomes dominant. At low concentrations, the second growth phase is no longer observable because the macrocyclic molecules are not able to stack into columns with the length and/or concentration needed for further packing. This also suggests that at low but aggregating concentrations, individually dissolved columnar stacks dominate.

### Individually dissolved columns in solution

The likely presence of dispersed stacks of **3a** in CHCl_3_ was probed with steady-state fluorescence anisotropy at 25 °C (see the ESI†). At 10 nM, a concentration at which **3a** remains fully aggregated as shown by its emission spectrum (Fig. S15†), the aggregate of **3a**, assumed to be a rotating “sphere”, has a diameter of 3.0 nm that is surprisingly close to that of **3a** with side chains included. A plausible explanation is that the revealed diameter reflects the rotation of dispersed stacks of **3a** around their long axes. In solution, only the self-spin of the cylindrical stacks is detected because such spin is much faster than the tumbling of the stacks around directions perpendicular to their long axes. Based on the data from fluorescence anisotropy and a refined model involving cylindrical stacks, the stacks of **3a**, at 10 nM, have an average of six macrocyclic molecules (see the ESI†).

### Columnar assembly in the solid state

**Fig. 5:**
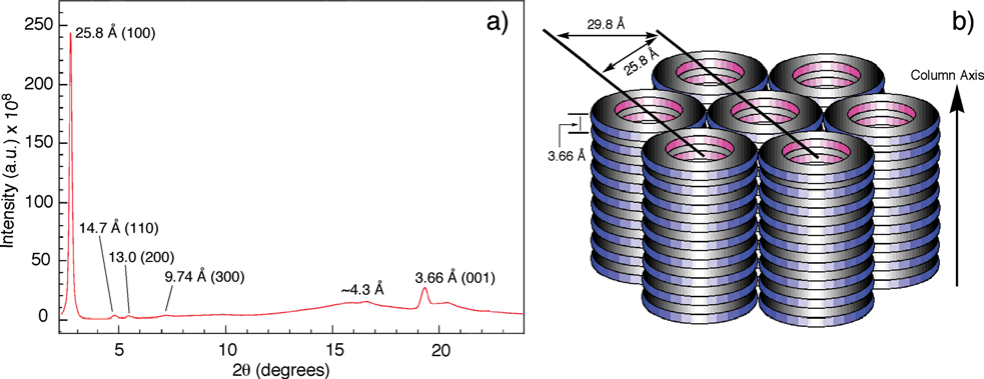
(a) Diffractogram of the solid sample of **3a**. (b) Schematic drawing of the columnar packing of **3a** and the hexagonal lattice. The hexagonal lattice parameter *a* is 29.8 Å.

The columnar assembly of **3a** was confirmed by XRD analysis on a solid sample prepared by drop-casting a solution in chloroform onto a glass plate. The obtained diffractogram contains a very intense peak at 25.8 Å that overshadows other peaks (Fig. 5). The 25.8 Å reflection and those at 14.7 Å, 13.0 Å, and 9.74 Å, with ratios of *d*-spacings being 
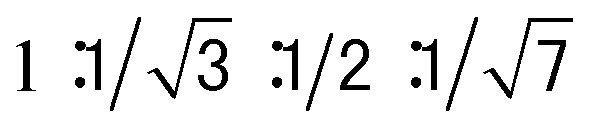
 (Fig. 5a), are typical of columnar stacks of disc-like molecules that further packed on a hexagonal (col_h_) lattice (Fig. 5b).^27^ Based on the 25.8 Å peak, the hexagonal lattice parameter *a*, *i.e.*, the diameter of the cylindrical stacks of **3a**, was calculated to be 29.8 Å. The diameter determined by XRD agrees with that obtained from fluorescence anisotropy, which demonstrates that **3a** stacks into cylindrical assemblies in both solution and the solid state. Moreover, a prominent peak at 3.66 Å, typical of π–π stacking, is observed. This peak can be attributed to the interplanar reflection between macrocyclic backbones within a column. Applying Scherrer's equation^28^ to this 3.66 Å reflection leads to a correlation length of 22 nm that corresponds to ∼60 continuously stacked macrocyclic units, which demonstrates the remarkable long-range ordering of the macrocycles within a column.

### Transmembrane transport of proton (H^+^)

**Fig. 6:**
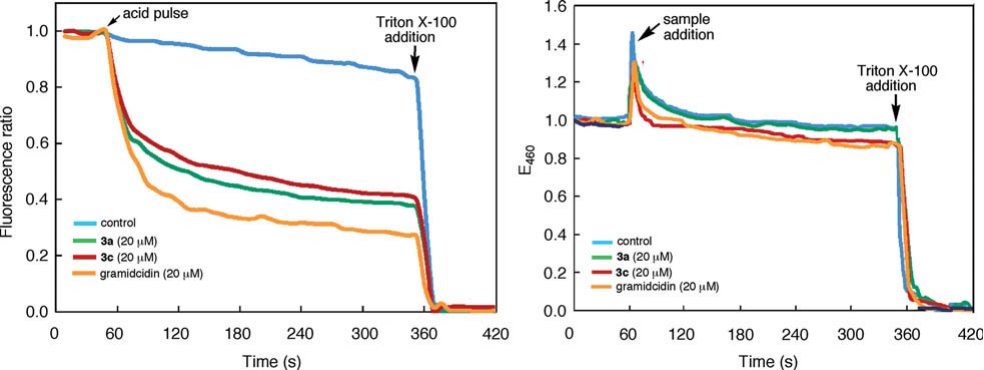
(a) Time-dependent changes in the (a) ratio of the emission intensities of 8-hydroxypyrene-1,3,6-trisulfonate (HPTS, 0.1 mM) encapsulated inside large unilamellar vesicles (LUVs) of 1-palmitoyl-2-oleoyl-*sn-glycero*-3-phosphocholine (POPC). Solutions of LUVs (KCl 145 mM, HEPES 100 mM, pH 7.0) were first mixed with **3a**, **3c**, gramicidin (added from 1 mM stock solutions in THF), or THF (control) and then incubated for 2 min, followed by a HCl (2 M) pulse. The ratio of emission intensities at 510 nm by exciting at 450 nm and 405 nm respectively was monitored over 5 min; (b) the fluorescence intensity of *N*-(ethoxycarbonylmethyl)-6-methoxyquinolinium bromide (MQAE) encapsulated inside LUVs of POPC. Solutions of Cl^–^ free LUVs (10 mM MQAE, potassium gluconate 100 mM, HEPES 100 mM, pH 7.4) were first incubated with KCl (100 mM) in HEPES (100 mM, pH 7.4) buffer for 1 min. Stock solutions of **3a**, **3c**, gramicidin (1 mM in THF) and THF (control) were added to monitor the change of emission intensity at 460 nm (*λ*_ex_ = 354 nm) for 5 min. The LUVs were ruptured by adding 200 μ(L of 1×X lysis buffer (1.55 mM Triton X-100 in pH 7.0 Tris-EDTA) (for (a)) or 200 μ(L of Triton X-100 (3.1 mM in H_2_O) (for (b)).

The tubular assembly of **3**, with a non-deformable hydrophilic internal pore of ∼8 Å across, could serve as transmembrane channels when partitioning into lipid bilayers. Thus, a solution of large unilamellar vesicles (LUVs) enclosing the pH-sensitive dye HPTS was mixed with **3a** and then subjected to an extravesicular acid (HCl) pulse. As shown in Fig. 6a, the decrease of fluorescence emission from the entrapped HPTS in the presence of **3a** is similar to that caused by gramicidin, a well-known channel-forming peptide and is much faster than that of the control. Rupture of the LUVs upon adding Triton X-100, a nonionic surfactant, led to the nearly complete reduction of fluorescence emission. Under the same condition, macrocycle **3c** also led to the same reduction of fluorescence emission, suggesting that the observed increase in proton influx was mediated by the inner pores, rather than the side chains, of the tubular assembly.

The transport of anions, or the lack of which, through the nanopores of **3a** or **3c** was assessed by using LUVs enclosing MQAE, a chloride-sensitive fluorescence dye.^29^ It was found that, in the presence of a chloride gradient across the lipid bilayer, adding **3a**, **3c** or gramicidin failed to quench the fluorescence emission from the entrapped MQAE (Fig. 6b). As expected, rupturing the LUVs with Triton X-100 led to complete quenching of fluorescence emission from MQAE. These results demonstrate that the self-assembling pores of **3**, with numerous inward pointing amide oxygens, and thus being electrostatically negative, facilitated the transport of cations while impeded the passage of anions.

## Conclusions

Our study shows that the self-assembly of macrocycles **3** is remarkably strong, which affords a robust nanotubular motif that persists in both solution and the solid state. With their sub-nm inner pores, the tubular assemblies of **3** should be of wide use in constructing various nanostructures. For example, with their high stability and tunable solvent-compatibility (by adjusting side chains), the tubular stacks of **3** bode well for developing various mass-transporting channels when partitioned into biological membranes, as exemplified by the cation-transporting capabilities of **3a** and **3c**. The persistent nanotubular assemblies of **3** may also serve as a reliable supramolecular motif for fabricating nanoporous membranes, *e.g.*, by blending with synthetic polymers. The high propensity of the tubular assemblies of **3** for parallel packing may lead to the next-generation membranes consisting of densely packed sub-nm pores. Furthermore, methods adopted in this study should be of general value for analysing hierarchical processes of other self-assembling systems, especially those involving extended or infinite stacks, which remains a major challenge.
