# Supplementary material for: Extremely strong tubular stacking of aromatic oligoamide macrocycles
Source: Chem Sci. 2014 Sep 16;6(1):152–7. doi: 10.1039/c4sc02380c (PMC5424541; doi:10.1039/c4sc02380c)
Supplement: Supplementary file 1 [file SC-006-C4SC02380C-s001.pdf]

Electronic Supplementary Material (ESI) for Chemical Science.
This journal is © The Royal Society of Chemistry 2014

Extremely Strong Tubular Stacking of Aromatic Oligoamide Macrocycles

Mark A. Kline,[†,‡] Xiaoxi Wei,[†,‡] Ian J. Horner,[†] Rui Liu,[†] Shuang Chen,[¶] Si Chen,[#] Ka Yi Yung,[†] Kazuhiro Yamato,[†] Zhonghou Cai,[#] Frank V. Bright,[†] Xiao Cheng Zeng,[¶] and Bing Gong*,[†,§]

[†]Department of Chemistry, The State University of New York at Buffalo, Buffalo, New York 14260, United States. [¶]Department of Chemistry, University of Nebraska-Lincoln, Lincoln, Nebraska 68588, United States. [#]X-ray Science Division, Argonne National Laboratory, 9700 South Cass Avenue, Argonne, IL 60439. [§]College of Chemistry, Beijing Normal University, Beijing 100875, China

Suppllementary Information

Table of Contents

1. General Experimental Methods S2
2. Dynamic Light Scattering (DLS) S2
3. Fluorescence Spectroscopy S3
4. Fluorescence Anisotropy S4
5. X-ray diffraction (XRD) S6
6. Computational Study S6
7. Supporting Figures S8
8. Table S1 S20
9. References S21

1. General Experimental Methods

Compounds **3a-3d** were synthesized as reported before.[1] Chemical grade reagents were used without further purification. The 1X lysis buffer mentioned in the legend of Figure 6a was provided by Gene and Cell Technologies, Inc., CA. *^{1}H NMR spectra* were recorded at 500 MHz on a Varian INOVA spectrometer. Chemical shifts were expressed in parts per million (δ) using tetramethylsilane (TMS) or residual solvent protons as internal standards (^{1}H: chloroform δ7.26 ppm; DMSO δ2.50 ppm). *Diffusion-ordered spectroscopy (DOSY)* experiments were performed on a Varian Inova 500 MHz spectrometer under regulated temperature (298 K), with a 5 mm probe. The pulse sequence employed was a bipolar pulse pair simulated echo (BPPSTE). Additional parameters: gradient strength array has 15 increments from 3 to 94% of the maximum gradient strength in a linear ramp, diffusion gradient length is set to 2 ms, diffusion delay is 100 ms.

2. Dynamic Light Scattering (DLS)

DLS measurements were performed on a Brookhaven 90plus Particle Analyzer. The wavelength of laser is 532 nm. Fluorescence spectra were recorded on a Perkin Elmer LS55 luminescence spectrometer.

The hydrodynamic diameters of the assembly of macrocycles **3a** and **1a** in mixed solvents containing DMF and $CHCl_3$ were measured at room temperature. A total of three experiments were performed per data set and averaged.

Two stock solutions of **3a** (1 mM), each prepared in DMF and $CHCl_3$ respectively, were filtered immediately through a 0.45-μm filter to remove dust and debris, and left to stand for 15-min before each series of measurements. The first measurement started with 3 mL of **3a** (1 mM) in DMF, followed by removing a pre-calculated aliquot from the DMF solution that had been measured, to which the same volume of the **3a** stock solution in $CHCl_3$ was added to result in the desired volume percent $CHCl_3$ while the concentration of **3a** was maintained at 1 mM. This procedure was repeated by removing the needed volume of the measured solution out of the cuvette, followed by adding the same volume of the **3a** stock solution in $CHCl_3$ to the cuvette, until measurements were completed on all the compositions of $CHCl_3$.

To let the aggregational process reach equilibrium, a 15-min rest period after mixing was allowed before each measurement was performed. The hydrodynamic diameters of the 1 mM solutions of **1a** at different $CHCl_3$ volume ratios were similarly measured after the samples have been rested for 15 minutes and 24 hours.

The viscosity of mixed solvents containing different portions of DMF and $CHCl_3$ was measured by an in-house computational program written via a similar protocol.[1] The refractive index of DMF and $CHCl_3$ mixtures were calculated via Wiener equation.[2,3]

3. Fluorescence Spectroscopy

Fluorescence measurements in parallel with DLS. To further probe the effect of $CHCl_3$ on the aggregation of **3a** and **1a**, a set of fluorescence experiments were performed analogously to the DLS studies of **1a** and **3a** at the reduced concentration of 1 μM. Once samples were tested with DLS, they were quickly examined with fluorescence (within 5 minutes of the DLS measurements) spectroscopic measurements. Fluorescence emission spectra of **1a** and **3a** in mixed solvents containing different ratios of $CHCl_3$ in DMF were obtained (λ_{ex} = 282 nm, $Slit_{ex}$ = 4 nm, $Slit_{em}$ = 5 nm) with a scan speed of 100 nm/min.

*Recording fluorescence emission spectra of **1a** and **3a** at various concentrations in $CHCl_3$*. Stock solutions of **1a** and **3a** (1 μM) in spectroscopic grade $CHCl_3$ were prepared one hour before each round of experiments. Samples were prepared by simple dilution to reach the required concentrations. Fluorescence spectra of **1a** and **3a** in $CHCl_3$ at different concentrations were obtained (λ_{ex} = 282 nm, $Slit_{ex}$ = 8 nm, $Slit_{em}$ = 10 nm) with a scan speed of 100 nm/min.

*Estimating the "dimerization" constant of macrocycle **3a***. The calculation was performed based on a "monomer-dimer" equilibrium that is made at the extremely low concentration of 1 pM (aggregated, assuming to be mainly dimers), with a conservative estimate of 10% dissociation of the dimers. Such an assumption is reasonable because at 0.1 pM, macrocycle **3a** exists mainly as monomers.

Recording fluorescence excitation spectra. Excitation spectra of **3a** in spectroscopic grade solvents were collected at both λ_{em} = 350 nm and λ_{em} = 450 nm with background subtraction ($Slit_{ex}$ = 4 nm, $Slit_{em}$ = 5 nm).

*Times course of aggregation at 1 μM of **1a** and **3a** in solvents with different ratios of $CHCl_3$ in DMF*. The times course of aggregation at 1 μM of **1a** and **3a** was assessed based on the following steps: Stock solutions of both **1a** and **3a** in DMF (198 μM) were prepared and diluted to 1 μM into mixed solvents with various ratios of $CHCl_3$ pre-mixed with DMF (spectroscopic grade). The stock solutions were prepared 1 hour before measurements, which were performed immediately after dilution. Aggregational times course was followed by recording emission intensities at λ = 450 nm ($Slit_{ex}$ = 8 nm, $Slit_{em}$ = 10 nm over 1200 minutes).

Times course of aggregation at significantly reduced concentrations. Solutions of **1a** and **3a** were prepared in DMF at various concentrations. Each stock solution was prepared one hour before measurements. The measurements were performed immediately after dilution by adding DMF stock solution to spectroscopic grade $CHCl_3$. The final volume ratio of $CHCl_3$ and DMF of each experiment is 99.9:0.1. Aggregational times course was followed by recording emission intensities at λ = 450 nm ($Slit_{ex}$ = 8 nm, $Slit_{em}$ = 10 nm over 1200 mins). Fluorescence emission spectra of the samples were collected immediately following the above procedure (λ_{ex} = 282 nm, $Slit_{ex}$ = 8 nm, $Slit_{em}$ = 10 nm).

All time-resolved intensity decays were measured by using an IBH model 5000 W SAFE time-correlated single photon counting (TCSPC) fluorescence lifetime instrument. A 280 nm light emitting diode (Nano LED) served as the excitation source. Emission was recorded at 450 nm (32 nm bandpass). All experiments were conducted until there were at least 10^4 counts in the peak multichannel analyzer channel. The typical time resolution for an experiment was between 0.04 and 0.05 ns/channel and 1024 total channels were used.

The TCSPC traces were analyzed by using Globals WE (Globals Unlimited), a commercially available nonlinear least-squares analysis software package, and evaluate the reduced χ^2, residuals and autocorrelation traces to determine the best fit model. The solvent blank was paramatized within a Global analysis strategy to account for its contribution to the sample signal.

4. Fluorescence Anisotropy

All steady-state fluorescence measurements were performed by using a SLM-AMINCO model 8100 spectrofluorimeter (SLM Instruments, Inc.) with a 450 W Xe arc lamp excitation

source. The sample temperature was maintained by using a temperature bath (Brookfield model TC-620D). The excitation wavelength was maintained at 282 nm. For emission spectra and steady-state fluorescence anisotropy experiments, the excitation and emission spectral bandpasses were 4 and 8 nm and 8 and 32 nm, respectively.

The stacking of **3a** in solution was probed with steady-state fluorescence anisotropy at 25 ^{o}C in $CHCl_3$. By monitoring the 450-nm emission band (λ_{ex} = 282 nm) that serves to indicate aggregation in $CHCl_3$, at 10 nM, a concentration at which **3a** engages in ground-state aggregation as shown by its emission spectrum (Figure S15), an excited-state fluorescence lifetime (τ) of 2.35 ± 0.03 ns and a steady-state fluorescence anisotropy (r) of 0.0529 ± 0.0013 were found for the aggregate formed by **3a**. Besides, the fundamental fluorescence anisotropy (r_0) of **3a** (10 nM) was determined to be 0.1167 ± 0.0023 in glycerol at 0 °C.

Based on Perrin equation:

$$r = r_0/(1 + \tau/\theta) \qquad (1)$$

and the experimentally determined r_0, r, and τ values, the rotational correlation time θ of the aggregate of **3a** was calculated to be 1.94 ns.

The rotational correlation time is in turn given by:

$$\theta = \eta V/RT \qquad (2)$$

where η is solvent viscosity, T temperature in K, R the gas constant, and V the molar volume of the rotating unit (i.e., the aggregate) being examined. Based on the value of θ, the molar volume (V) of the aggregate of **3a** was found to be 14.76 nm^3. If a spherical shape is assumed for the aggregate, a diameter of 3.0 nm, a value very close to the diameter of the macrocyclic molecule, was obtained for the rotating "sphere" formed by **3a**, which suggests that the measured rotational correlation time for **3a** reflects the spin of the cylindrical stacks of **3a** around their long axes.

Instead of assuming a spherical shape for the aggregate of **3a** 10 nM in $CHCl_3$, a model based on a cylinder consisting of stacked **3a** may allow the number of macrocyclic molecules

that form such stacks to be estimated. Given that the radius (r) of **3a** monomer is 14.9 Å (XRD data, Figure 5), and the molar volume of the aggregate of **3d** is 14.76 nm^3 (see above), based on the equation for the volume of a column:

$$V = h\pi r^2$$

The average height (h) of the stacks of **3a** is 2.12 nm, which, based on the stacking distance of **3a** in a column (3.66 Å, Figure 5), gives an average number of ~6 (5.79) molecules for the stacks of **3a**.

5. X-ray diffraction (XRD)

X-ray diffraction was recorded at the 2-ID-D beam line of the Advanced Photon Source at Argonne National Laboratory using 10.1 Kev radiation (λ = 1.2275 Å) on a Newport 6-circle (Kappa) diffractometer. X-ray diffraction was measured with a QUAD-RO CCD detector (Princeton Instruments, Trenton, NJ) placed behind the specimen. The detector-to-sample distance was varied based on the angular range of the required measurement. Because the limited field views of the CCD detector, usually full diffraction rings were measured for those relatively small angle diffractions, and partial rings were measured for those large angle diffractions. The diffraction intensities of individual reflections were integrated along their rings and plotted along 2θ angles.

6. Computational Study

Figure S16(a) shows the molecular structure of macrocycle **1e** optimized at the level of M06-2X/6-31G(d) implemented in the Gaussian 09 software package,[4] where the molecular axis is highlighted by a blue arrow. To understand the intermolecular interaction between stacked macrocycles, two such molecules are stacked in parallel to form different dimers as the function of rotation (stacking) angle (θ) and interlayer distance (r). The stacking angle and interlayer distance are respectively defined as the angle between two molecular axes and the distance between the center of mass of each monomer (see Figure S16(b)). First, the dimer with initial r = 3.3 Å varies as a function of θ from 0° to 60°. These dimers are

optimized by using the Kohn-Sham formulation of density-functional theory (DFT) and the Gaussian plane-wave (GPW) method[5] implemented in the CP2K software package.[7] The Becke-Lee-Yang-Parr (BLYP) functional[8,9] is employed for structural optimization. The core electrons are described by the Goedecker-Teter-Hutter (GTH) norm-conserving pseudopotential,[10,11] and the wave functions of valence electrons are expressed by the combination of the polarized double-ξ quality Gaussian basis[12] and a plane-wave basis set (with an energy cutoff of 280 Ry). To better describe the long-range electron correlations that are responsible for the van der Waals (vdW) interactions between two monomers, the Grimme dispersion corrected (DFT-D3) method[13] is adopted. After geometry optimization of the dimers, the single-point energies of these dimers are computed using the more accurate M06-2X/6-31G(d) method. The computed binding energies (see Figure S17) are computed with basis set superposition error (BSSE) correction[14,15] implemented in the Gaussian 09 software package.[4] As shown in Figure S17, the dimer with r = 3.486 Å and θ = 60.5° has the largest binding energy of about -49.77 kcal/mol. The strong binding is most likely due to the strong π–π interaction as well as local dipole interaction between two monomers.

7. Supporting Figures

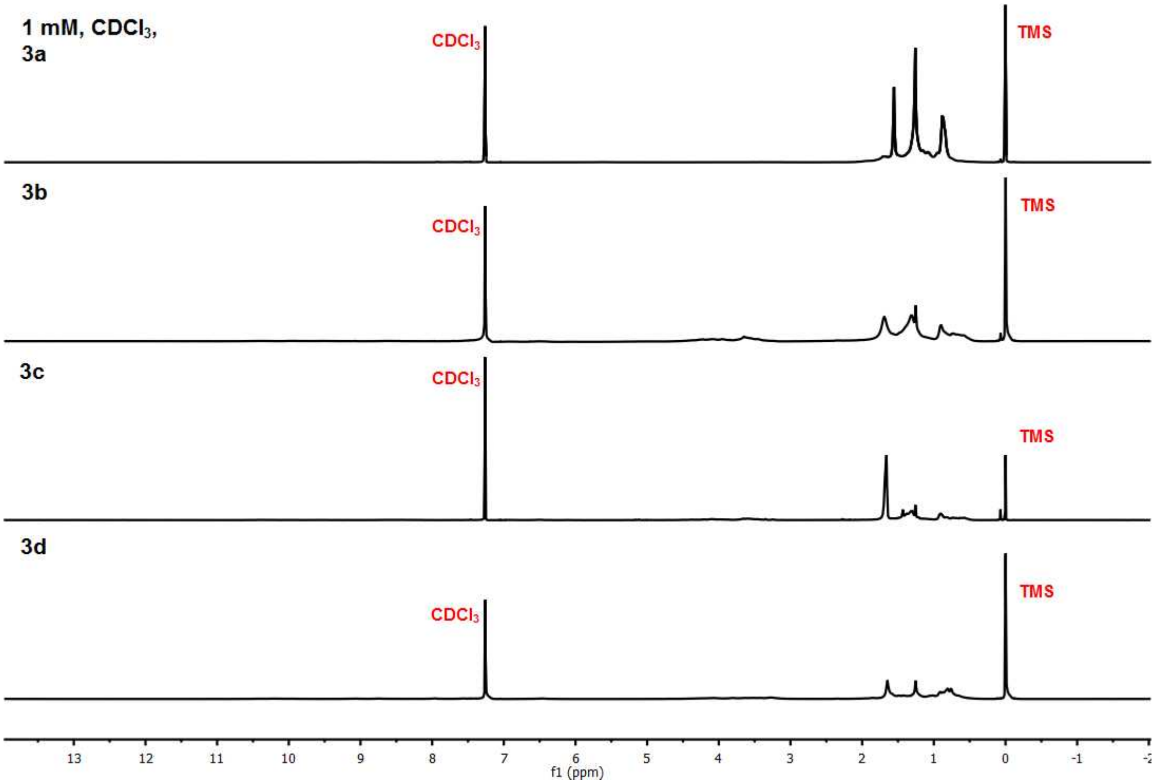

Figure S1. ^{1}H NMR spectra of macrocycles **3a-d** (1 mM in $CDCl_3$, 500 MHz, 25 ^{o}C).

(a)

(b)

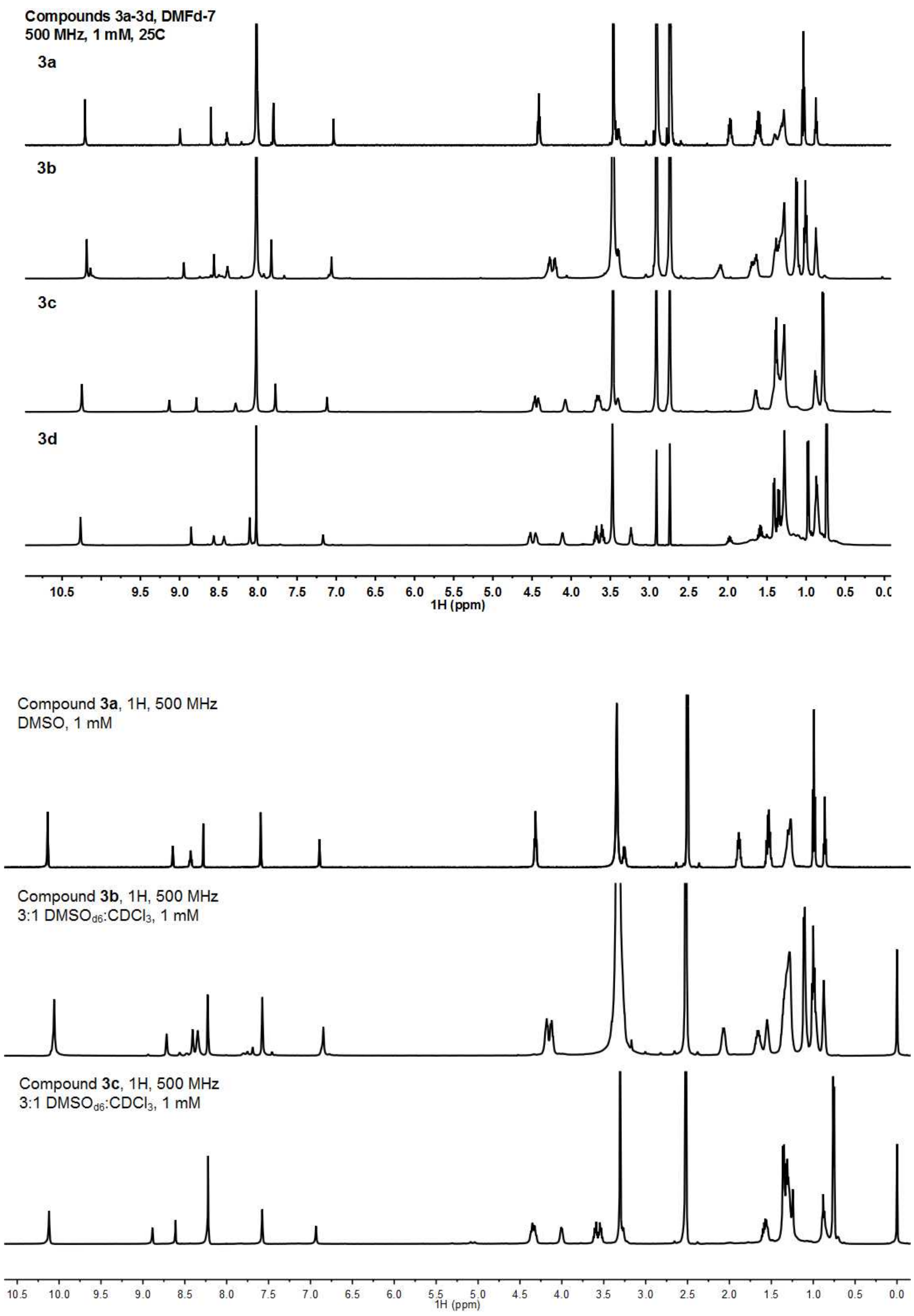

Figure S2. Full-range ^{1}H NMR spectra of (a) **3a-3d** in DMF-d_7 and (b) **3a-3c** in DMSO-d_6 or DMSO-d_6/$CDCl_3$ (3/1, v/v).

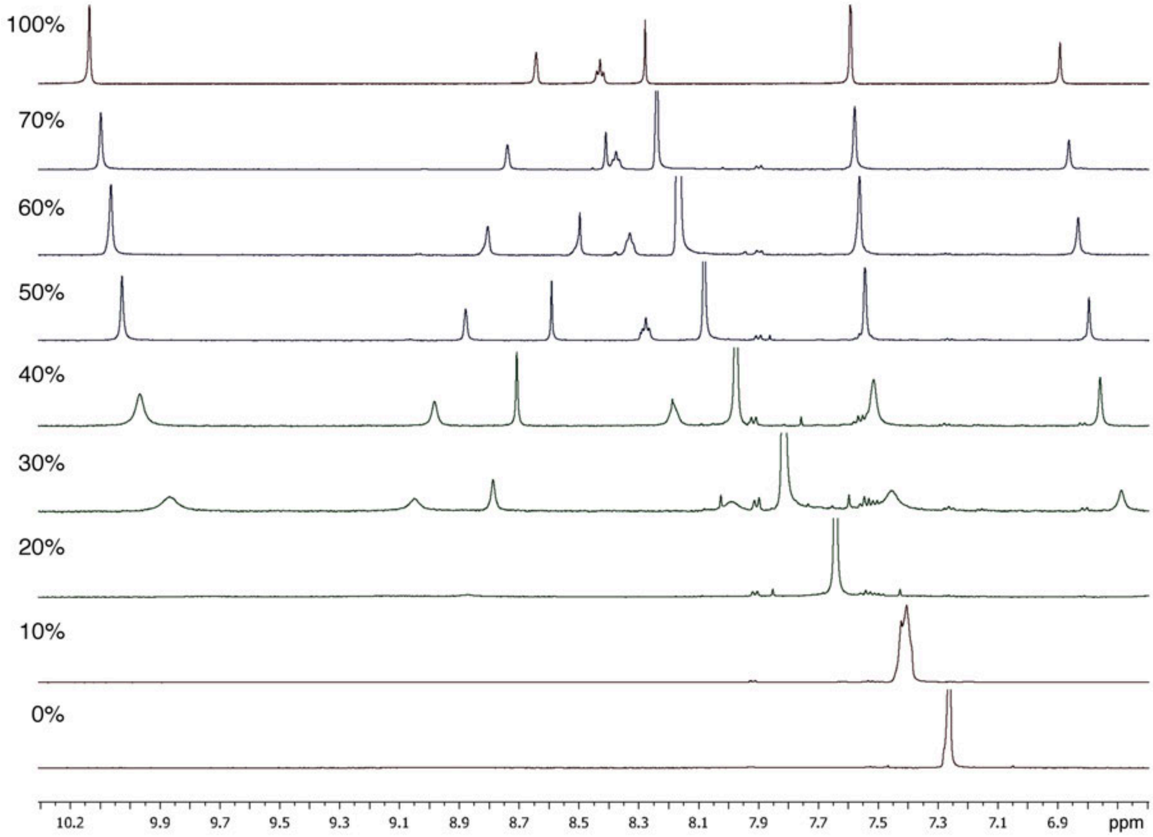

Figure S3. Partial ^{1}H NMR (500 MHz, 25 ºC) spectra of **3a** (1 mM) in solvents containing various volume percent DMSO-d_6 in $CDCl_3$. All NMR samples were prepared from the same stock solutions of **3a** in DMSO-d_6 and $CDCl_3$.

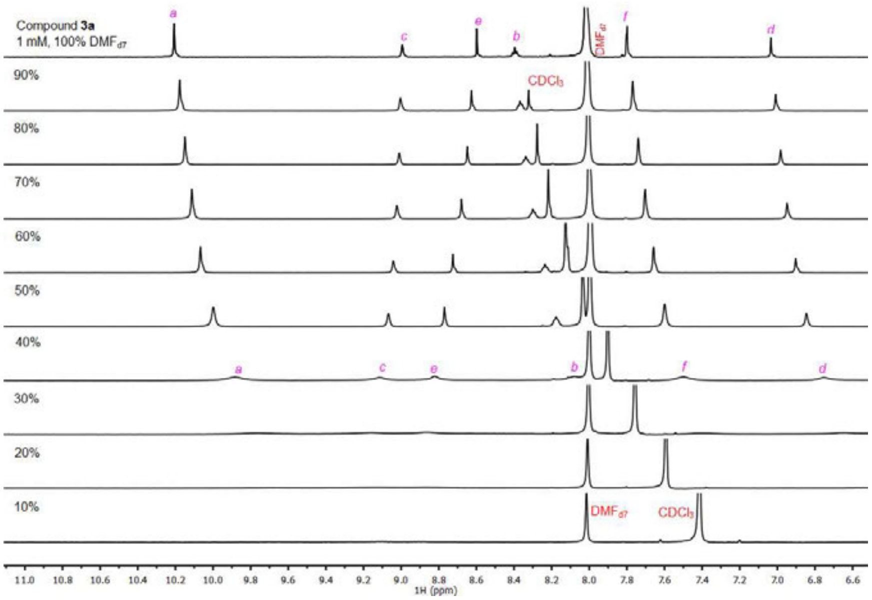

Figure S4. Partial ^{1}H NMR (500 MHz, 25 ^{o}C) spectra of **3a** (1 mM) in solvents containing various volume percent DMF-d_7 in $CDCl_3$.

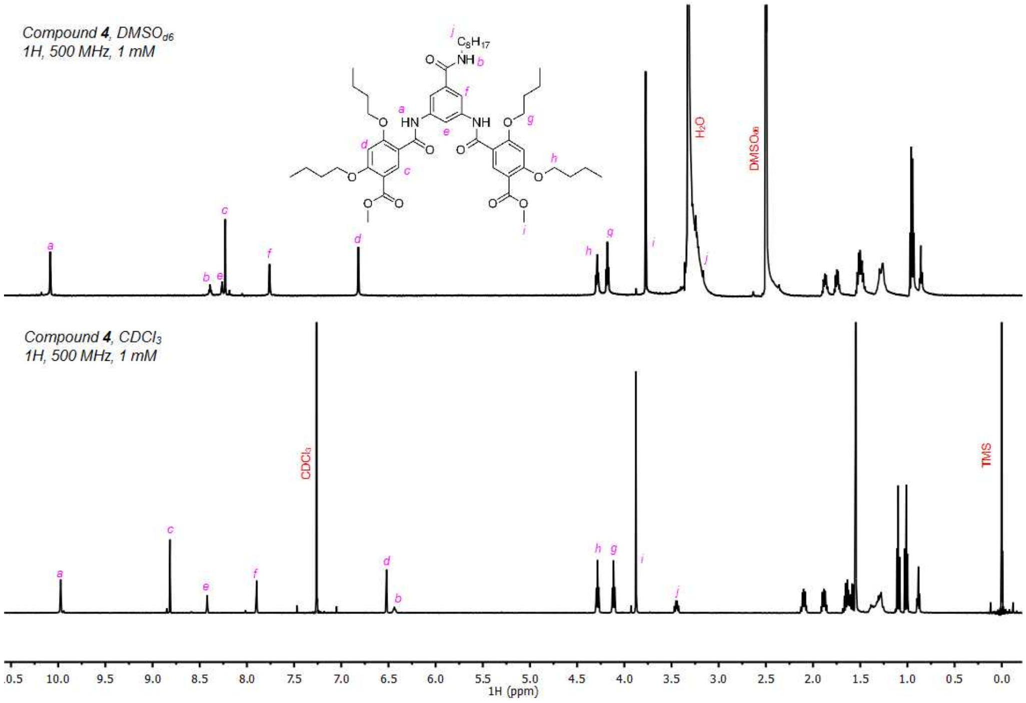

Figure S5. ^{1}H NMR (500 MHz, 25 ^{o}C) spectra of **3a** (1 mM) in DMSO-d_6 (top) and $CDCl_3$ (bottom).

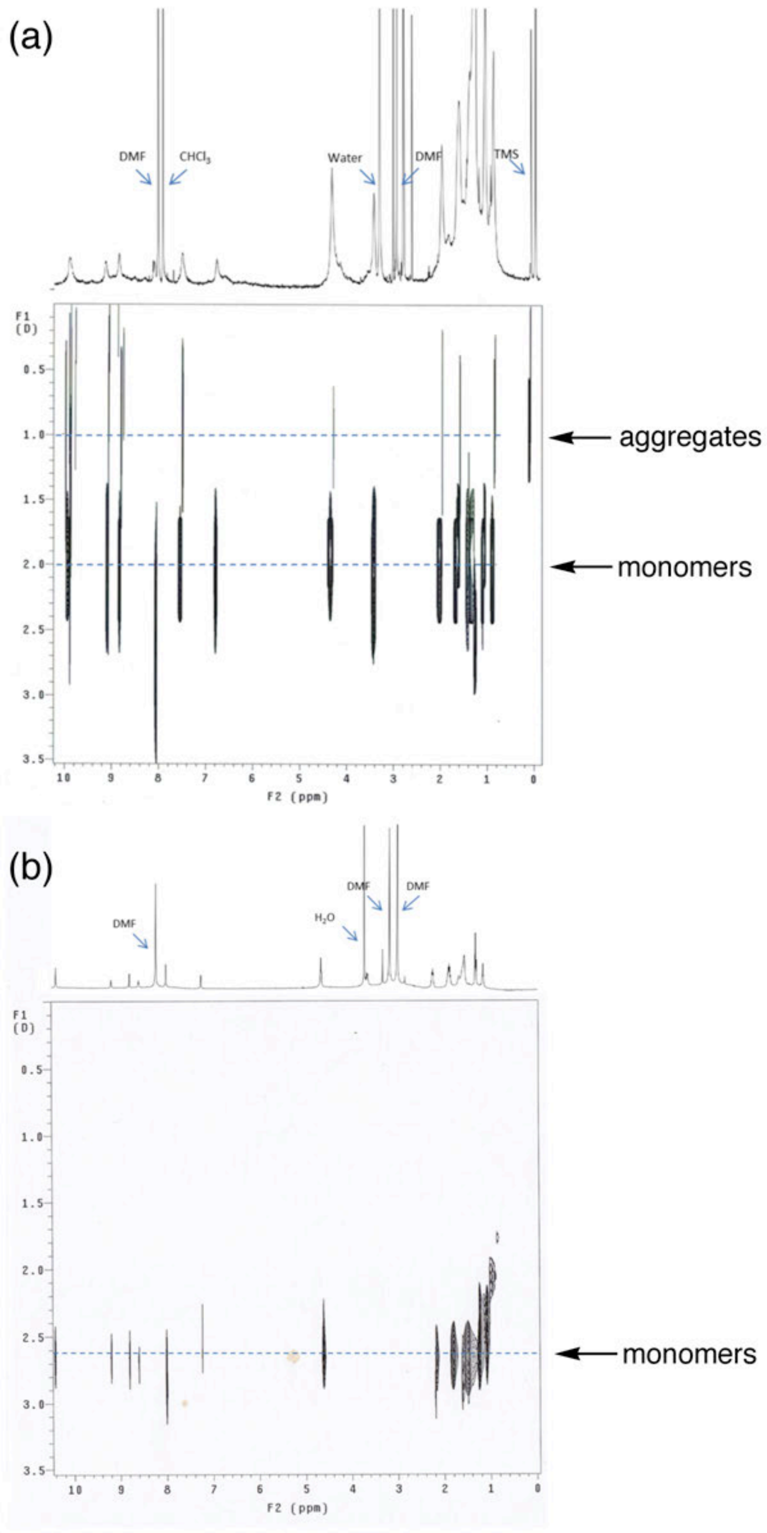

Figure S6. Partial DOSY spectra of **3a** (1mM) (a) in $CDCl_3$ 40% DMF-d_7 and (b) in 100% DMF-d_7.

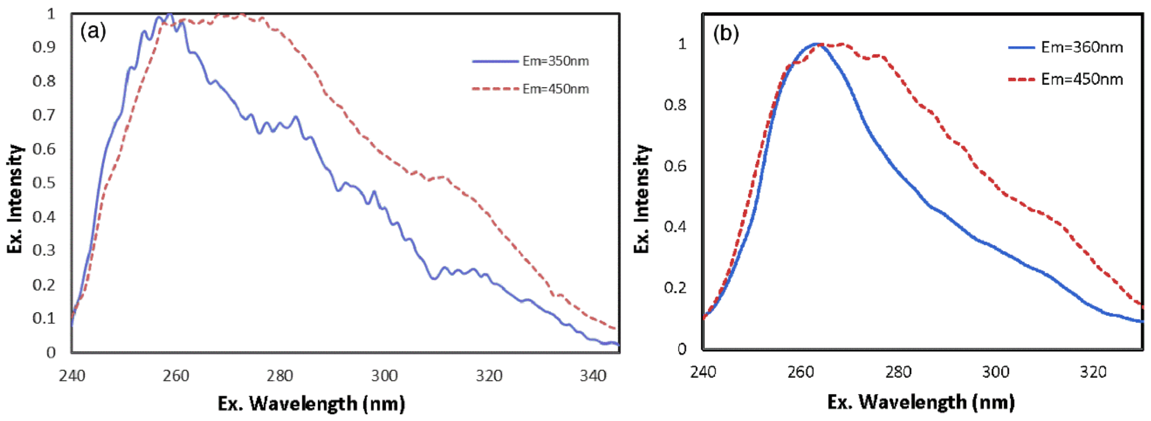

Figure S7. Fluorescence excitation spectra of (a) 125 nM, (b) 0.1 pM of **3a** in $CHCl_3$ monitored at 350 nm or 360 nm (blue) and 450 nm (red) with background subtraction [$Slit_{ex}$ = 4 nm, $Slit_{em}$ = 5 nm].

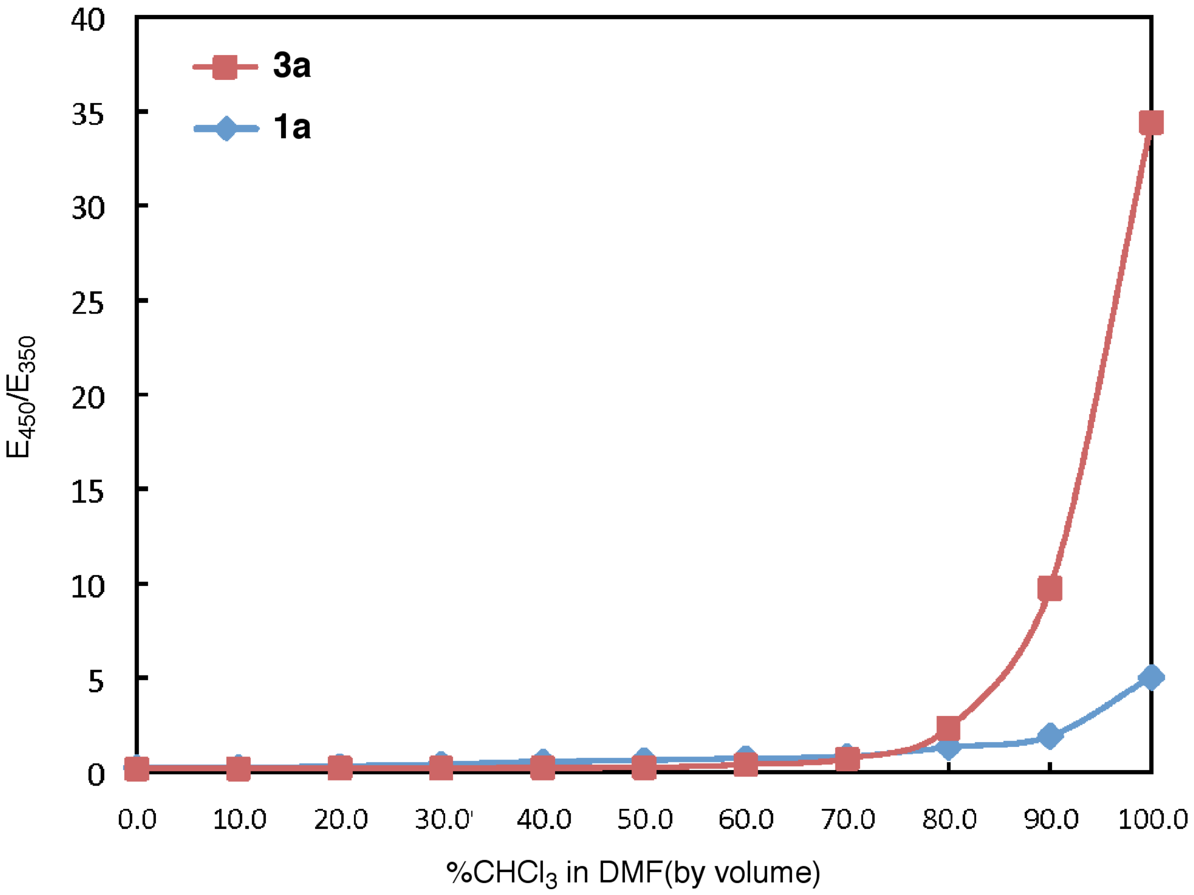

Figure S8. Ratio of the 450-nm and 350-nm emission bands, E_{450}/E_{350}, which serves as an indicator for the aggregation of **1a** (1 μM, blue) and **3a** (1 μ M, red), versus volume percent of $CHCl_3$ in DMF at 25 °C.

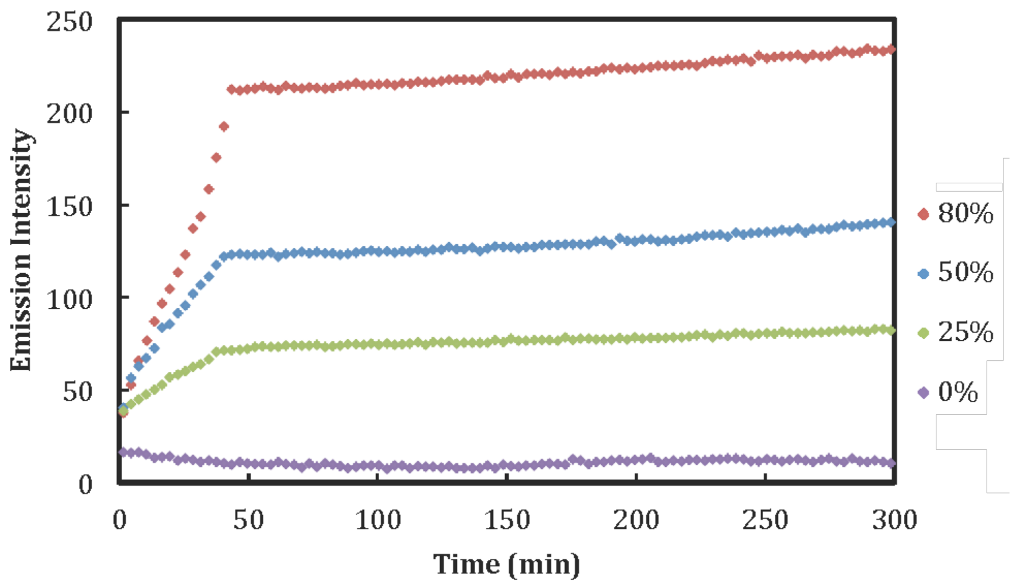

Figure S9. Time courses for the fluorescence emission intensities (at 450 nm) of **3a** (1 μM) at 450 nm vs volume% $CHCl_3$ in DMF.

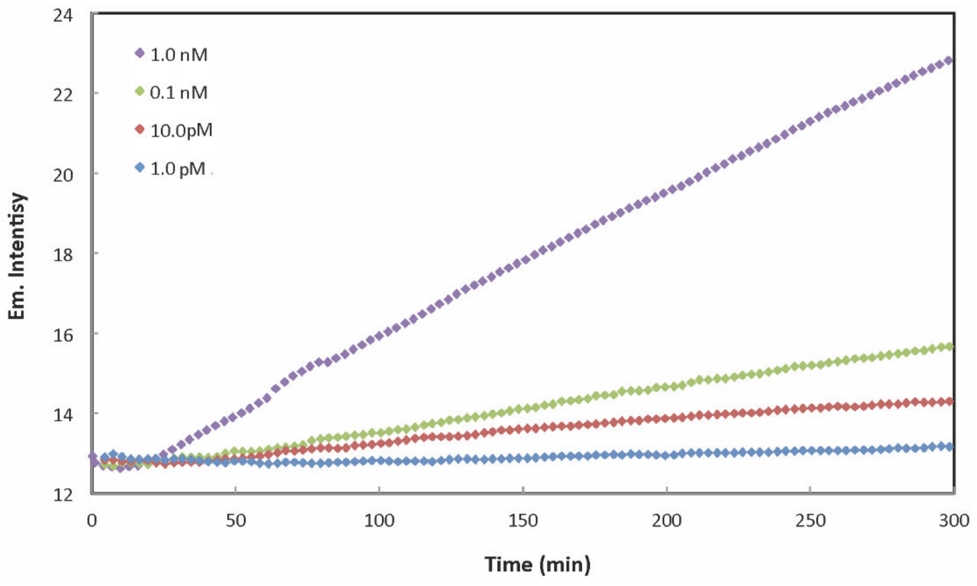

Figure S10. Time courses for the fluorescence emission intensities (at 450 nm) of **3a** at various concentrations in 99.9% $CHCl_3$ and 0.1% DMF.

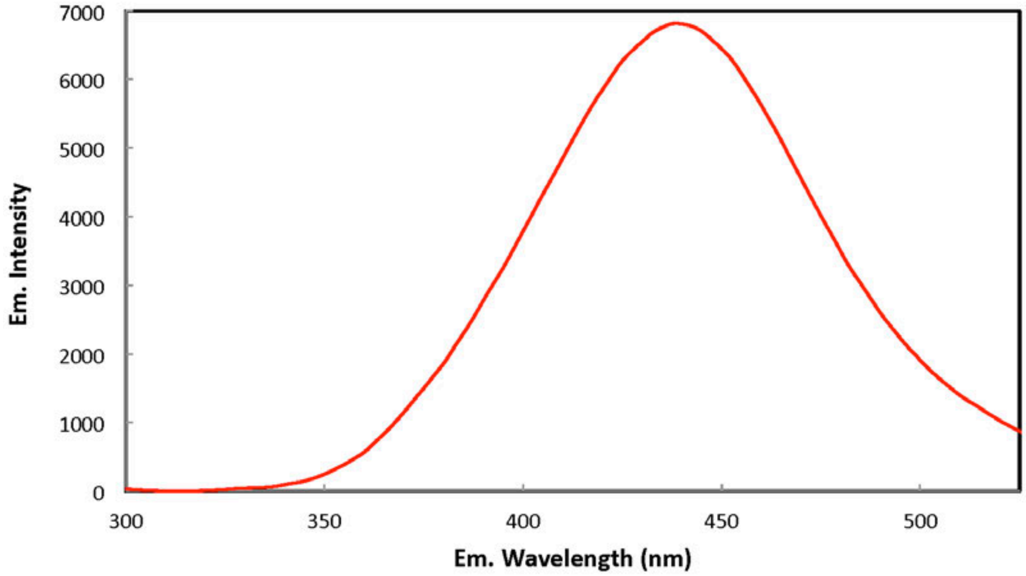

Figure S11. Emission spectrum of **3a** (10 nM) in the mixed solvent containing 99.9% $CHCl_3$ and 0.1% DMF.

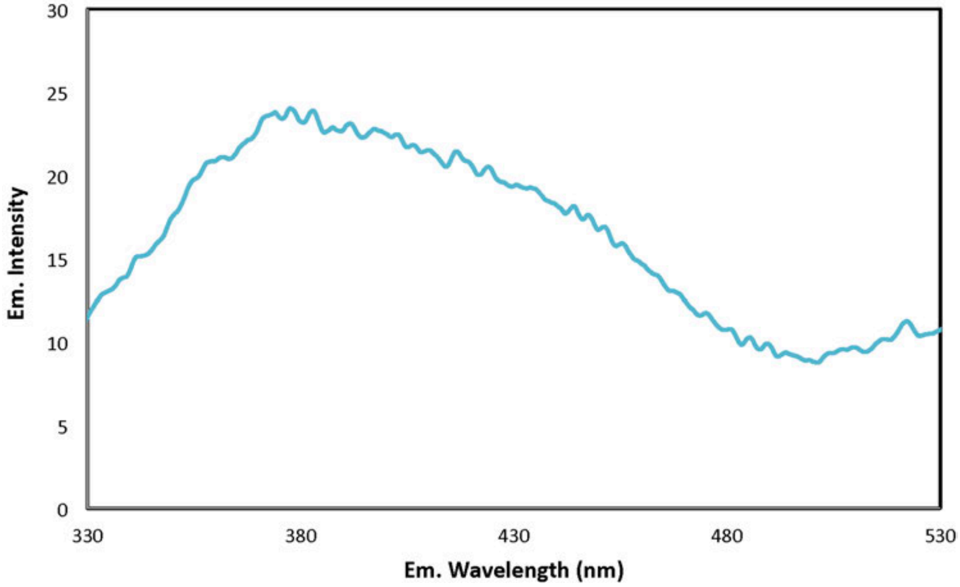

Figure S12. Emission spectrum of **3a** (1 pM) in the mixed solvent containing 99.9% $CHCl_3$ and 0.1% DMF.

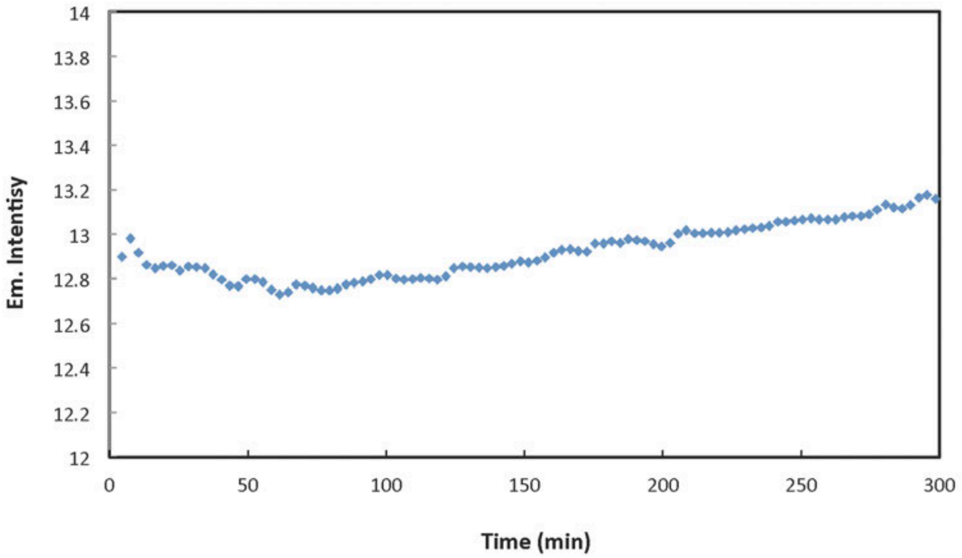

Figure S13. Time course for the fluorescence emission intensities (at 450 nm) of **3a** (1 pM) in the mixed solvent containing 99.9% $CHCl_3$ and 0.1% DMF.

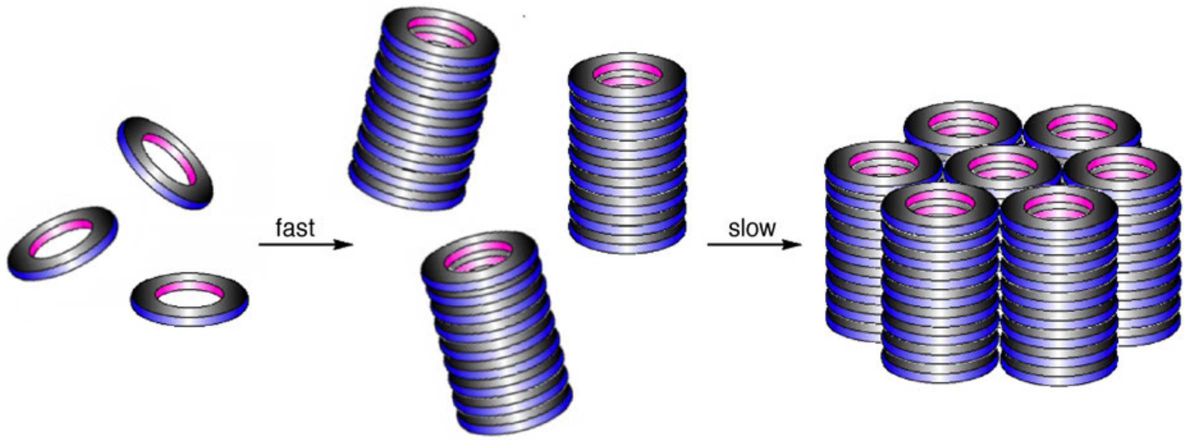

Figure S14. The two phases of the aggregation of macrocycles **3**: the rapid stacking of the macrocyclic molecules into columns and the slow packing of individually dissolved columns into bundles.

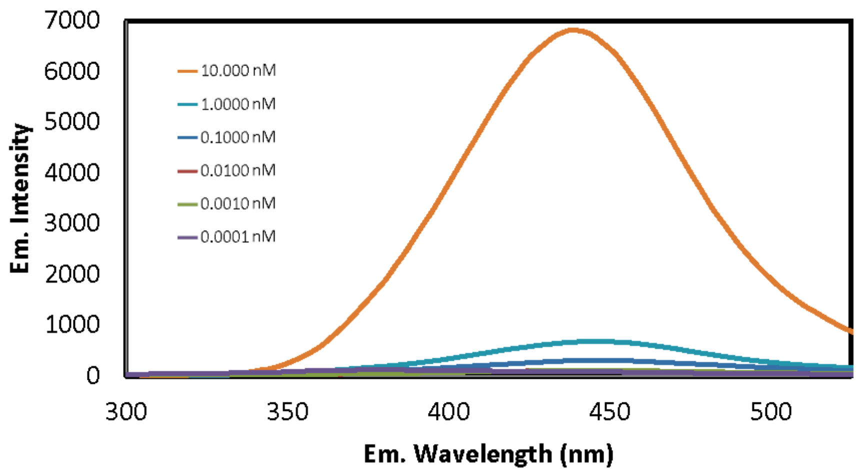

Figure S15. Emission spectra of **3a** in $CHCl_3$ at various concentrations.

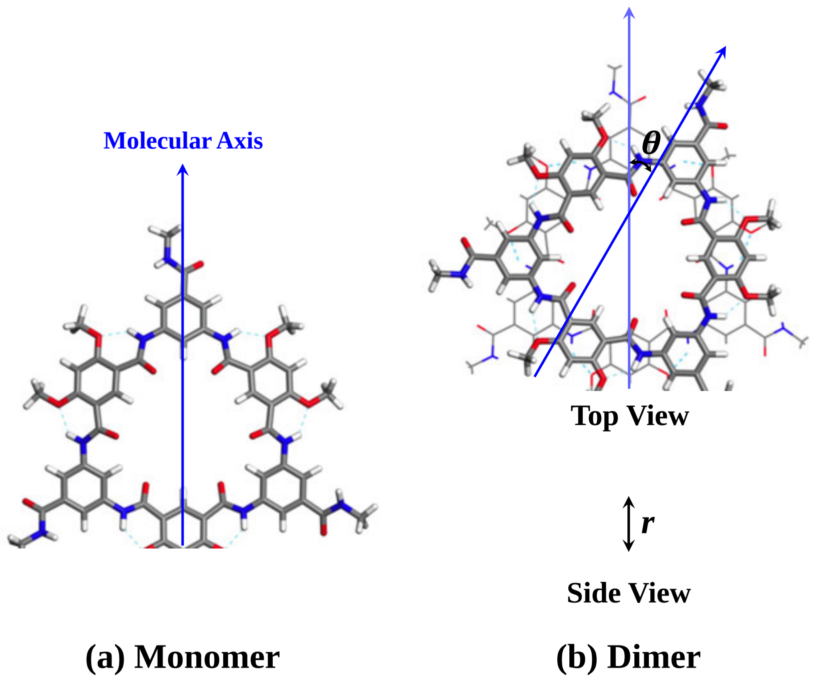

Figure S16. (a) M06-2x/6-31G(d)-optimized monomer and (b) BLYP-D3/GTH optimized dimer. The molecular axis is highlighted by a blue arrow. The rotation angle (θ) and interlayer distance (r) are respectively defined as the angle between two molecular axes and the distance between the center of mass of each monomer.

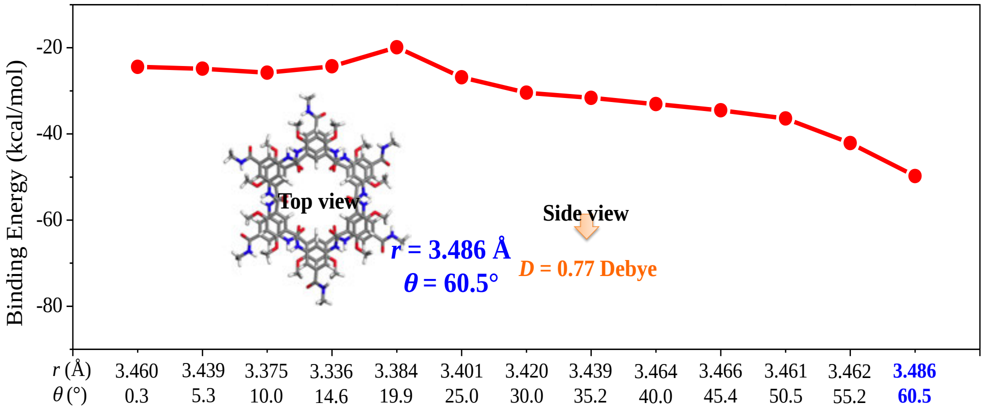

Figure S17. Computed binding energies with BSSE correction for dimers with different interlayer distance (r) and rotation angle (θ). The dimer with r = 3.486 Å and θ = 60.5°, whose structure is highlighted in insets, has the strongest binding energy. The dimers are optimized at the BLYP-D3/GTH level of theory and the binding energies are computed at the M06-2X/6-31G(d) level of theory.

8. Table S1

Ratios of normalized fluorescence emission of **1a** and **3a** (1 μM) at 450 nm and 350 nm (E_{450}/E_{350}) vs volume% of $CHCl_3$ in DMF.

Volume% $CHCl_3$ in DMF	**E_{450}/E_{350}**	
	**1a**	**3a**
0.0%	0.23385	0.19019
10.0%	0.24498	0.20186
20.0%	0.33124	0.22100
30.0%	0.42024	0.22631
40.0%	0.56238	0.24138
50.0%	0.64068	0.26017
60.0%	0.74397	0.40204
70.0%	0.84293	0.72311
80.0%	1.48594	2.34813
90.0%	1.87874	9.75216
100.0%	2.07645	34.39988

9. References

1. Kline, M.; Wei, X. X.; Gong, B. *Org. Lett.* **2013**, *15*, 4762.
2. Maples, R. E. *Petroleum Refinery Process Economics*, 2nd Ed., Wilfried Pennwell Books, Tulsa, Okla, 2000.
3. Wiener, O. *Leiprig. Ber.* **1910**, *62*, 256.
4. Heller, W. *J. Phys. Chem.* **1965**, 69, 1123.
5. Frisch, M. J.; Trucks, G. W.; Schlegel, H. B.; Scuseria, G. E.; Robb, M. A.; Cheeseman, J. R.; Scalmani, G.; Barone, V.; Mennucci, B.; Petersson, G. A.; Nakatsuji, H.; Caricato, M.; Li, X.; Hratchian, H. P.; Izmaylov, A. F.; Bloino, J.; Zheng, G.; Sonnenberg, J. L.; Hada, M.; Ehara, M.; Toyota, K.; Fukuda, R.; Hasegawa, J.; Ishida, M.; Nakajima, T.; Honda, Y.; Kitao, O.; Nakai, H.; Vreven, T.; Montgomery, J. A.; Peralta, J. E.; Ogliaro, F.; Bearpark, M.; Heyd, J. J.; Brothers, E.; Kudin, K. N.; Staroverov, V. N.; Kobayashi, R.; Normand, J.; Raghavachari, K.; Rendell, A.; Burant, J. C.; Iyengar, S. S.; Tomasi, J.; Cossi, M.; Rega, N.; Millam, J. M.; Klene, M.; Knox, J. E.; Cross, J. B.; Bakken, V.; Adamo, C.; Jaramillo, J.; Gomperts, R.; Stratmann, R. E.; Yazyev, O.; Austin, A. J.; Cammi, R.; Pomelli, C.; Ochterski, J. W.; Martin, R. L.; Morokuma, K.; Zakrzewski, V. G.; Voth, G. A.; Salvador, P.; Dannenberg, J. J.; Dapprich, S.; Daniels, A. D.; Farkas, O.; Foresman, J. B.; Ortiz, J. V.; Cioslowski, J.; Fox, D. J. Gaussian 09, Revision A.02; Gaussian, Inc.: Wallingford, CT, 2009.
6. Lippert, G.; Hutter, J. R.; Parrinello, M. *Mol. Phys.* **1997**, *92*, 477.
7. VandeVondele, J.; Krack, M.; Mohamed, F.; Parrinello, M.; Chassaing, T.; Hutter, J. *Comput. Phys. Commun.* **2005**, *167*, 103.
8. Becke, A. D. *Phys. Rev. A* **1988**, *38*, 3098.
9. Lee, C.; Yang, W.; Parr, R. G. *Phys. Rev. B* **1988**, *37*, 785.
10. Goedecker, S.; Teter, M.; Hutter, J. *Phys. Rev. B* **1996**, *54*, 1703.
11. Hartwigsen, C.; Goedecker, S.; Hutter, J. *Phys. Rev. B* **1998**, *58*, 3641.
12. VandeVondele, J.; Hutter, J. *J. Chem. Phys.* **2007**, *2007*, 114105.
13. Grimme, S.; Antony, J.; Ehrlich, S.; Krieg, H. *J. Chem. Phys.* **2010**, 132, 154104.
14. Boys, S. F.; Bernardi, F. *Mol. Phys.* **1970**, 19, 553.
15. Simon, S.; Duran, M.; Dannenberg, J. J. *J. Chem. Phys.* **1996**, 105, 11024.
